# Comparison of CRISPR-Cas9/Cas12a Ribonucleoprotein Complexes for Genome Editing Efficiency in the Rice Phytoene Desaturase (*OsPDS*) Gene

**DOI:** 10.1186/s12284-019-0365-z

**Published:** 2020-01-21

**Authors:** Raviraj Banakar, Mollie Schubert, Michael Collingwood, Christopher Vakulskas, Alan L. Eggenberger, Kan Wang

**Affiliations:** 1 Department of Agronomy, Iowa State University, Ames, IA USA; 2 Crop Bioengineering Center, Iowa State University, Ames, IA USA; 3 Present Address: Department of Plant and Microbial Genomics, University of Minnesota, St Paul, MN 55108 USA; 4 Integrated DNA Technologies, Coralville, IA USA

**Keywords:** CRISPR, Cas9, Cas12a, Rice, Ribonucleoproteins, Synthetic guide RNAs

## Abstract

**Background:**

Delivery of CRISPR reagents into cells as ribonucleoprotein (RNP) complexes enables transient editing, and avoids CRISPR reagent integration in the genomes. Another technical advantage is that RNP delivery can bypass the need of cloning and vector construction steps. In this work we compared efficacies and types of edits for three Cas9 (WT Cas9 nuclease, HiFi Cas9 nuclease, Cas9 D10A nickase) and two Cas12a nucleases (AsCas12a and LbCas12a), using the rice phytoene desaturase (PDS) gene as a target site.

**Findings:**

Delivery of two Cas9 nucleases (WT Cas9, and HiFi Cas9) and one Cas12a nuclease (LbCas12a) resulted in targeted mutagenesis of the PDS gene. LbCas12a had a higher editing efficiency than that of WT Cas9 and HiFi Cas9. Editing by Cas9 enzymes resulted in indels (1–2 bp) or larger deletions between 20-bp to 30-bp, which included the loss of the PAM site; whereas LbCas12a editing resulted in deletions ranging between 2 bp to 20 bp without the loss of the PAM site.

**Conclusions:**

In this work, when a single target site of the rice gene *OsPDS* was evaluated, the LbCas12a RNP complex achieved a higher targeted mutagenesis frequency than the AsCas12a or Cas9 RNPs.

## Findings

Clustered regularly interspaced short palindromic repeats-CRISPR associated (CRISPR-Cas) is an adaptive immune system in prokaryotes that protects against invading bacteriophages by performing cleavage of their DNA (Horvath and Barrangou 2010; Garneau et al. 2010; Gasiunas et al. 2012). CRISPR systems were later adapted to precisely edit the genomes of many species including plants (Nekrasov et al. 2013). Successful examples of editing in different plant species include rice, corn, wheat, soybean, and tomato (Mikami et al. 2016; Lee et al. 2019; Kelliher et al. 2019; Biswas et al. 2019; Svitashev et al. 2016; Gil-Humanes et al. 2017; Okada et al. 2019; Cai et al. 2019).

Three main types of CRISPR systems have been described thus far, Types I, II and III. CRISPR-Cas9 and CRISPR-Cas12a from the Type II CRISPR systems are two major nucleases that have been exploited to edit plant genomes (Nekrasov et al. 2013; Svitashev et al. 2015; Kim et al. 2017). The CRISPR-Cas9 system from *Streptococcus pyogenes* recognizes an NGG protospacer adjacent motif (PAM) to create double strand breaks upstream of the PAM site, whereas the CRISPR-Cas12a (formerly Cpf1) system recognizes the TTTV PAM to create double strand breaks downstream of the PAM recognition site (Svitashev et al. 2015; Kim et al. 2017). Therefore, these two proteins are of use for gene editing in different genomic contexts as Cas9 can be used for editing GC-rich regions and Cas12a can be used for editing AT-rich regions. In addition, there is a considerable difference between the results of Cas9 and Cas12a cleavage, in which Cas9 creates blunt ended DNA breaks near the PAM site whereas Cas12a generates staggered DNA breaks distal to the PAM site (Svitashev et al. 2015; Kim et al. 2017). Hence, comparing these proteins is of interest for different genome editing purposes.

There are mutant variants of Cas9 proteins from *Streptococcus pyogenes* available such as High Fidelity SpCas9 (HiFi Cas9) and Cas9 nickases (SpCas9 D10A and SpCas9 H840A) (Schiml et al. 2014; Shen et al. 2014; Vakulskas et al. 2018). In comparison to WT Cas9, HiFi Cas9 exhibits reduced off-target cleavage (Vakulskas et al. 2018). Cas9 nickase mutants (D10A and/or H840A) can be used simultaneously to introduce a DSB with overhangs provided that multiple guides are used to position DNA nicks in the proper PAM out orientation, where the guides target opposite strands of DNA with their PAMs facing away from each other. It has been demonstrated that the use of paired D10A nickases allow for the reduction of off-target editing in comparison to WT Cas9 (Ran et al. 2013; Cho et al. 2014). While mutant forms of Cas9 have been created to alter or improve its function, Cas12a enzymes from different prokaryotic species, typically *Acidaminococcus sp*. BV3L6 (AsCas12a) and *Lachnospiraceae bacterium* ND2006 (LbCas12a), have been used to maximize genome editing in living cells (Jacobsen et al. 2019; Pu et al. 2019). Cas12a proteins from different species exhibit markedly different cleavage properties, most notably LbCas12a functions better at lower temperatures which is ideal for delivery into ectothermic organisms such as zebrafish or plants (Kim et al. 2017; Tang et al. 2017; Malzahn et al. 2019).

CRISPR reagents can be delivered into plants by *Agrobacterium* mediated T-DNA transfer (Char et al. 2017; Lee et al. 2019), biolistic plasmid delivery (Svitashev et al. 2016; Gil-Humanes et al. 2017; Hamada et al. 2018) or biolistic delivery of ribonucleoprotein (RNP) complexes (Svitashev et al. 2016; Liang et al. 2018, 2019). Using purified Cas9 or Cas12a proteins and chemically synthesized guide RNAs eliminates the possibility of continuous expression and ensures that these reagents are present transiently and thus minimizing the opportunity for off-target editing to occur (Svitashev et al. 2016).

**Fig. 1:**
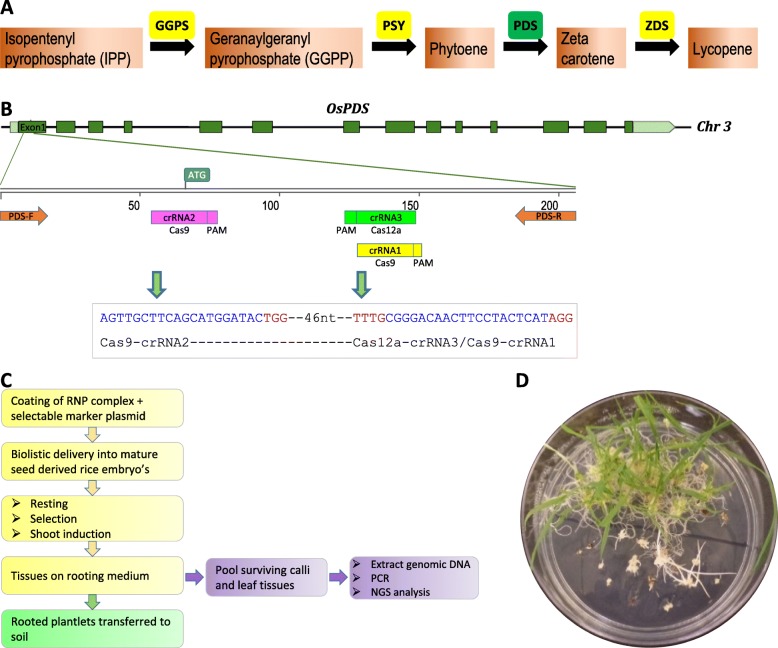
Choice of Carotenoid biosynthesis pathway to evaluate CRISPR-Cas nucleases. **a** Carotenoid biosynthesis pathway in rice. Phytoene desaturase (PDS) is a single copy gene involved in the synthesis of zeta-carotene from phytoene. GGPS, geranylgeranyl pyrophosphate synthase; PSY, phytoene synthase; PDS, phytoene desaturase; ZDS, zeta-carotene desaturase. **b** Schematic diagram showing OsPDS gene structure, OsPDS-Exon1 and relative position of crRNA1/2 for CRISPR-Cas9 and crRNA3 for CRISPR-Cas12a. PDS-F and PDS-R, forward and reverse primer pair for PCR and NGS analysis. **c** Flowchart showing RNP complex delivery and editing efficiency comparison analysis. **d** Regenerating green and albino rice plantlets on hygromycin containing rooting medium

Phytoene desaturase (PDS) catalyzes the conversion of phytoene into zeta carotene (Fig. 1a), a key step in the carotenoid biosynthetic pathway (Mann et al. 1994). PDS is encoded by a single copy gene in rice (*PDS*, Os03g0184000), which has 14 exons and 13 introns. Bi-allelic knock out of this gene results in an albino phenotype in callus tissue or in plant leaves, making PDS a preferred target for the evaluation of genome editing reagents. In this work, the *PDS* gene was used as a target to evaluate five different CRISPR-Cas nucleases. These enzymes were WT Cas9, HiFi Cas9, Cas9 D10A nickase, AsCas12a and LbCas12a.

To maximize the likelihood for effective gene knock out, we chose to target DNA sequences that are proximal to the start codon. crRNAs for all the CRISPR-Cas nucleases target the antisense strand of the *PDS* first exon (Fig. 1b, Table 1). Because the purpose of this experiment was to compare the efficacies of Cas9 and Cas12a nucleases for editing genomes, we identified DNA sequences that could be targeted by both Cas9 and Cas12a. A 36 nt crRNA containing 20 nt of unique targeting sequence (CGGGACAACTTCCTACTCAT, Cas9 crRNA1) for Cas9 enzymes (WT Cas9, HiFi Cas9, and Cas9 D10A), and a 41 nt crRNA that contains 21 nt of targeting sequence (CGGGACAACTTCCTACTCATA, Cas12a crRNA3) for Cas12a enzymes (AsCas12a and LbCas12a) were chosen to target the same DNA sequence (Fig. 1b, Table 1). The Cas9 D10A nickase was used with paired crRNAs, the crRNA1 used with WT Cas9 and HiFi Cas9, and crRNA2 (AGTTGCTTCAGCATGGATAC), which targeted the antisense strand at the start codon 53-bp upstream of crRNA1. It should be noted that due to the requirement of having to be in close proximity to the site targeted by both Cas9 and Cas12a, it was necessary to have the crRNA2 targeting the same strand as crRNA1 rather than the preferred targeting of the opposite strand with the crRNAs having the ‘PAM-out’ orientation. The crRNAs have a similar GC content, 50% for crRNA1, 45% for crRNA2, and 47.6% for crRNA3. Base repeats can in some cases influence the secondary structure of crRNA as well as crRNA-DNA binding ability which can cause detrimental effects on total editing (Svitashev et al. 2016). Towards this end, crRNA1 and crRNA3 have four repeats in total with a maximum repeat of three bases (GGG) followed by three repeats of two bases (AA, TT and CC), whereas crRNA2 has three repeats in total (TT, TT and GG), with all of them being two base repeats. The unavoidable inclusion of these dinucleotide repeats could influence the results of our experiment and should be considered.

**Table 1:** crRNA and enzymes used in the experiment

Nuclease	crRNA ID	crRNA sequence^a^	PAM	GC %	Length (nt)
WT Cas9 / HiFi Cas9 / Cas9 D10A	crRNA 1	CGGGACAACTTCCTACTCAT	AGG	50.0%	20
Cas9 D10A	crRNA 2	AGTTGCTTCAGCATGGATAC	TGG	45.0%	20
AsCas12a / LbCas12a	crRNA 3	CGGGACAACTTCCTACTCATA	TTTG	47.6%	21

^a^Base repeats are underlined

The different CRISPR enzymes (WT Cas9, HiFi Cas9, Cas9 D10A nickase, AsCas12a and LbCas12a), along with their respective guide RNAs, were delivered as RNP complexes into 5-day-old mature seed derived rice embryos (Additional file 1, Fig. 1c). To select and enrich for transformed cells, plasmid pCAMBIA1301 (Roberts et al. 1996; GenBank: AF234297.1) was co-delivered along with the RNP molecules. pCAMBIA1301, widely used for rice transformation, is a plasmid construct carrying the plant selectable marker gene hygromycin phosphotransferase (*hpt*), which is driven by the 2X CaMV 35S promoter and terminated by the *nos* terminator.

For each RNP complex and pCAMBIA1301 DNA co-delivery experiment, 30 embryos were bombarded in duplicate. Bombarded embryos were cultured on media containing 50 mg/L hygromycin, and hygromycin resistant and proliferating callus pieces were identified. As shown in Table 2, hygromycin resistant (hyg^R^) putative transgenic callus lines were produced with different rates. Among the five enzymes, both WT Cas9 and HiFi Cas9 generated the highest number of hyg^R^ callus pieces, achieving a transformation frequency of 16.7%. Transformation frequencies for LbCas12a and Cas9 D10A were 11.7% and 6.7%, respectively. AsCas12a produced hygromycin-resistant callus lines, but none of them were able to produce roots. As often observed in the plant transformation process, not all of the herbicide or antibiotic resistant callus lines have ability to regenerate and produce roots. Therefore, it is likely that the differences in transformation rates amongst different nuclease experiments observed here were due to the quality of explants and the fluctuate nature of the biolistic transformation process.

**Table 2:** Summary of transformation frequencies and editing efficiency for five CRISPR-RNP/selectable marker plasmid co-delivery experiments

Nuclease	# embryos bombarded	# hygR lines on regeration	# putative lines to soil	Transformation frequency^a^	# lines analyzed by NGS	# lines edited	Editing efficiency^b^
WT Cas9	60	56	10	16.7%	56	2	3.6%
HiFi Cas9	60	34	10	16.7%	34	3	8.8%
Cas9 D10A	60	30	4	6.7%	30	0	0.0%
AsCas12a	60	34	0	0.0%	34	0	0.0%
LbCas12a	60	42	7	11.7%	31	10	32.3%

^a^Transformation frequency = # putative lines to soil / # embryo bombarded × 100

^b^Mutation frequency = # mutated lines / # lines analyzed by NGS × 100

Next generation sequencing (NGS) analysis was performed on proliferating hygromycin-resistant callus materials. Surviving tissues from the rooting media plates originating from individual embryos were treated as clones and pooled for DNA extraction (Fig. 1c). A total of 185 genomic DNA samples from putative transgenic rice callus pieces generated by the five RNP co-delivery experiments were analyzed by NGS analysis of sequence surrounding the target site (Table 2).

Table 2 summarizes the variation in the editing efficiencies observed among the enzymes tested. Editing efficiency was the highest for LbCas12a, with a total of 10 edited lines out of 31 analyzed (32.3% editing efficiency). WT Cas9 achieved a 3.6% editing efficiency, two edited lines out of 56 tested. HiFi Cas9 produced the second-best editing efficiency of 8.8%, with three lines out of 34 lines tested (Table 2). None of the analyzed callus lines from AsCas12a and Cas9 D10A nickase were edited (Table 2). These results show that one of two Cas12a nucleases (LbCas12a) and two of three Cas9 enzymes (WT Cas9 and HiFi Cas9) worked in this experiment. If it is assumed that the almost identical target DNA sequences at the same site did not affect results, LbCas12a appeared to be 8.7-fold more efficient over WT Cas9 and 3.6-fold more efficient than HiFi Cas9 at targeted mutagenesis.

**Fig. 2:**
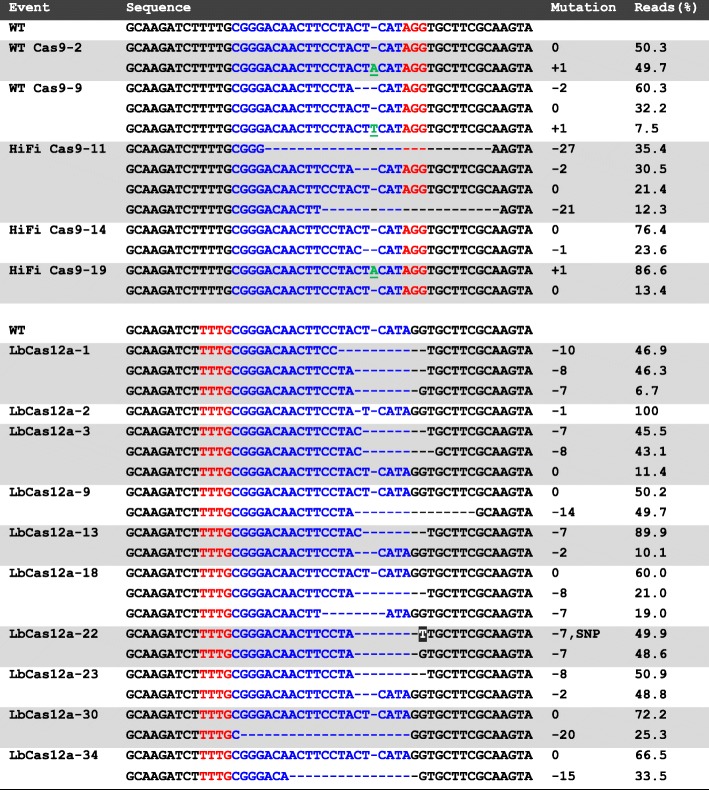
NGS analysis of rice lines generated from two Cas9 (WT Cas9 and HiFi Cas9) and one Cas12a (LbCas12a) RNP complex/selectable marker plasmid co-delivery. Total reads do not always add to 100% because small percentages of low frequency reads were excluded. These low frequency events are likely due to sequencing or alignment errors. Blue letters, target sequences in PDS exon 1; Red letters, PAM sequences; White letter in black box, substitution; Green letter with underscore, insertions; WT, wild type; SNP, single nucleotide polymorphism

Figure 2 presents NGS analysis results of 15 edited lines from the three enzymes that produced edits. Two WT Cas9 lines (WT Cas9–2 and − 9) had indels. WT Cas9–2 had 50.3% reads with no mutation and 49.7% reads with a 1-bp insertion 3 bp upstream of the PAM sequence. On the other hand, line WT Cas9–9 had 60.3% reads with a 2-bp deletion, 7.5% reads with a 1-bp insertion and 32.2% reads showing no mutation (Fig. 2). In the case of HiFi Cas9 we identified three lines with mutations (HiFi Cas9–11, − 14 and − 19, Fig. 2). HiFi Cas9–11 had a mixed population of mutations, with 21.4% reads with no mutations, 30.5% reads with a 2 bp deletion, 12.3% reads with a 21-bp deletion, and 35.4% reads with a 27-bp deletion. These extended deletions (21-bp and 27-bp) removed the PAM site. HiFi Cas9–14 and − 19 lines had a simpler editing pattern. HiFi Cas9–14 had a 1-bp deletion (23.6% of reads) that was 3 bp upstream of the PAM site with the remaining reads having no mutation. HiFi Cas9–19 had 86.6% reads showing a 1-bp insertion at 3 bp upstream of the PAM site and 13.4% of reads with no mutation (Fig. 2).

The LbCas12a co-delivery experiment produced ten mutated callus lines. These clones are named LbCas12a-1, -2, -3, -9, -13, -18, -22, -23, -30 and -34 (Fig. 2). All mutations appeared to be deletions of different sizes downstream of the PAM sequence. Of the ten edited lines, five lines (LbCas12a-1, − 2, − 13, − 22 and − 23) showed mutation reads only, namely, genomic DNAs from these lines did not contain non-mutated DNA sequence at the targeted location. These five lines displayed distinct colorless callus appearance as opposed to typical pale yellow morphology for hygromycin resistant callus lines. One event, LbCas12a-2, gave rise to albino seedlings. The other four mutant events did not regenerate to plantlets. One of the five lines (LbCas12a-2) had 100% reads showing a 1-bp deletion. Another line (LbCas12a-1) showed a mixed mutation population of three deletion sizes, 46.9% had a 10-bp deletion, 46.3% had an 8-bp deletion, and 6.7% had a 7-bp deletion. Three of the five complete mutation lines (LbCas12a-13, − 22 and − 23) carried two distinct mutation populations. LbCas12a-13 had a majority with a 7-bp deletion (89.9%) and a small population with a 2-bp deletion (10%). LbCas12a-22 had similar frequencies of a 7-bp deletion (48.6%) and the same 7-bp deletion plus a single nucleotide polymorphism at the deletion site (49.9%). LbCas12a-23 had 50.9% reads of 8-bp deletion and 48.8% reads of 2-bp deletion.

The other five LbCas12a lines appeared to have partial mutations. Two lines (LbCas12a-3 and − 18) had two types of mutations plus a non-mutated population. LbCas12a-3 had the majority reads of either 7-bp (45.5%) and 8-bp (43.1%) deletions and a small portion (11.4%) of non-mutant reads. LbCas12a-18, on the other hand, had majority reads of non-mutant (60%), but 21% reads of 8-bp deletion and 19% of 7-bp deletion. Three partial mutation lines (LbCas12a-9, − 30 and − 34) all seemed to have relatively large deletions, ranging from 14-bp to 20-bp. LbCas12a-9 showed equal rates of a 14-bp deletion (49.7%) and non-mutation (50.2%). In LbCas12a-30 line, around ¼ of reads were a 20-bp deletion (25.3%), while the majority (72.2%) were wild type sequences. Similarly, LbCas12a-34 had 1/3 reads of a 15-bp deletion (33.5%) and 2/3 were wild type (66.5%).

Unlike the Cas9 edited lines in which both insertions and deletions were present, nine out of ten edited LbCas12a lines had deletion mutations, with the other line (LbCas12a-22) having a SNP in nearly half of the reads and a 7-bp deletion predominating in the other reads. More importantly, none of the LbCas12a edited lines had lost a PAM site. It is known that the cutting sites of Cas9 are usually proximal to PAM site while that of Cas12a are distal from PAM site (Swarts and Jinek 2018). Except for lines LbCas12a-30 and − 34 in which deletions happened 1-bp and 7-bp, respectively, downstream of the PAM site, the majority of the edited lines had deletions starting from 11 to 15 bp downstream of PAM site. While sample size was limited, the majority of the Cas9 lines analyzed had either a 1-bp insertion or a 1–2 bp deletion, except for one line (HiFi Cas9–11) that had larger deletions. On the other hand, most of LbCas12a lines showed mutations with over 7-bp deletions.

Typically, hygromycin resistant callus after two rounds of selection is derived from a single cell of the infected rice embryo and is considered a putative transgenic callus line. This multi-cellular transgenic line is often called a clone, meaning that these cells are clonal for the introduced transgenes. However, callus tissue can be a mosaic for CRISPR generated mutations (Lee et al. 2019), thus sequencing results need to be interpreted with caution. Ideally, this type of analysis should be performed in callus-derived plantlets in which mono- or bi-allelic mutation types can be more readily assigned. We performed callus analysis instead of plant analysis in this work partially due to low regeneration rates of the experiments. However, we do recognize that line LbCas12a-2 appeared to have a uniform mutation population as it had 100% reads containing 1-bp indel. Several other lines appearing to have two populations of mutation with two sequencing reads occurring in an approximately 1:1 ratio. These lines are likely to produce homozygous and heterozygous mutant plants.

Though the sample size used in this work was too small to make a definitive comparison, our results suggest that LbCas12a is more efficient than the other nucleases tested. One caveat is that only one crRNA was tested for each Cas9 or Cas12a group, except for Cas9 D10 which required a second gRNA, and it is known that different crRNAs can affect nuclease efficiency. We tried to minimize this by having the Cas9 and Cas12a target sites almost completely overlap. The much higher editing efficiency of LbCas12a compared to AsCas12a is notable. It has been shown previously in soybean and tobacco protoplasts that LbCas12a has higher editing efficiency than AsCas12a when delivered as RNP molecules (Kim et al. 2017). Similarly, when tested in rice, Arabidopsis and corn LbCas12a does out-perform AsCas12a when these nucleases are delivered as plasmid molecules into protoplasts (Kim et al. 2017; Malzahn et al. 2019). More importantly, it must be noted that editing efficiency of Cas12a proteins is temperature dependent (Malzahn et al. 2019). These enzymes have been shown to have higher activity at the 37° temperature used for human cells, but the temperature used in our transformation experiments is 28 °C. It has been shown that the activity of LbCas12a is reduced by the lower temperatures used for plant transformation (Moreno-Mateos et al. 2017; Malzahn et al. 2019) and this is the likely reason. Hence, it is likely that lower temperature plus the lower overall activity of AsCas12a resulted in the absence of edits in this experiment.

Overall, we have shown the biolistic delivery of three different Cas9 and two different Cas12a RNPs in rice. Our results show that LbCas12a has a higher editing efficiency compared to the other enzymes at the one target sequence of one gene tested. Although the sample size is small, we did notice that the mutations generated by LbCas12a tended to have the PAM site preserved at the target site. This can be an additional feature of LbCas12a, which may be preferred to Cas9 for enabling subsequent re-editing at the target site. This work further illustrates need for careful consideration when selecting reagents for genome editing in rice and other plants.

## Supplementary information

**Additional file 1.** Materials and Methods.

## Data Availability

All information is provided in this article.

## Abbreviations

2.4.D: 2.4 Dichloro Phenoxy Acetic Acid
CRISPR: clustered regularly interspaced short palindromic repeats
PAM: Protospacer Adjacent Motif
NGS: next generation sequencing
SNP: single nucleotide polymorphism
