# Supplementary material for: Comparison of CRISPR-Cas9/Cas12a Ribonucleoprotein Complexes for Genome Editing Efficiency in the Rice Phytoene Desaturase (OsPDS) Gene
Source: Rice (N Y). 2020 Jan 21;13:4. doi: 10.1186/s12284-019-0365-z (PMC6973557; doi:10.1186/s12284-019-0365-z)
Supplement: Supplementary file 1 — Additional file 1. Materials and Methods. [file 12284_2019_365_MOESM1_ESM.pdf]

Supplementary information

Comparison of CRISPR-Cas9/Cas12a ribonucleoprotein complexes for genome editing efficiency in rice

Raviraj Banakar[1,2 #], Mollie Schubert[3], Michael Collingwood[3], Christopher Vakulskas[3], Alan L Eggenberger[1,2], and Kan Wang[1,2 *]

[1] Department of Agronomy, Iowa State University, Ames, IA, USA

[2] Crop Bioengineering Center, Iowa State University, Ames, IA, USA.

[3] Integrated DNA Technologies, Coralville, IA, USA

* Corresponding author:

Kan Wang

Email: kanwang@iastate.edu

[#] Current Address: Department of Plant and Microbial genomics, University of Minnesota, St Paul, MN 55108, USA.

Materials and Methods:

Choice of PDS1 gene and gRNA design

The single copy rice phytoene desaturase gene (Os03g0184000) was chosen as the target for CRISPR genome editing **(Fig. 1A)**. Three target sites in the first coding exon were chosen to compare the efficacy of five different CRISPR enzymes, CGGGACAACTTCCTACTCAT (targeted by Cas9 crRNA1), AGTTGCTTCAGCATGGATAC (targeted by Cas9 crRNA2) and CGGGACAACTTCCTACTCATA (targeted by Cas12a crRNA3) **(Fig. 1B)**. Cas9 crRNA1 was selected for use with Alt-R S.p. Cas9 nuclease and Alt-R S.p. HiFi Cas9 nuclease. Cas9 crRNA1 and Cas9 crRNA2 were chosen for use with Alt-R S.p. Cas9 D10A nickase. Cas12a crRNA3, which almost completely overlapped with Cas9 cRNA1, was designed for use with Alt-R A.s. Cas12a, and LbCas12a to allow for direct comparison of Cas9 and Cas12a enzymes in the same sequence location.

CRISPR proteins, crRNA and tracrRNA molecule synthesis

Alt-R S.p. Cas9 Nuclease V3, Alt-R HiFi S.p. Cas9 Nuclease V3, Alt-R S.p. Cas9 D10A Nickase V3, Alt-R A.s. Cas12a V3, and LbCas12a proteins were obtained from Integrated DNA Technologies (IDT), Coralville, IA, USA. Cas9 crRNA1, Cas9 crRNA2, Cas12a crRNA3, and tracrRNA molecules were chemically synthesized at IDT. The crRNAs contained the 20-21 nt guide sequence along with the constant region required for each protein tested. All reagents (enzymes, crRNA and tracrRNA) were shipped to Iowa State University in dry ice and reagents were stored at -20°C until further use.

CRISPR-RNP complex formation

On the day of CRISPR-RNP delivery to plant cells, 2 nmol crRNA and 5 nmol tracrRNA were dissolved in 40 µL and 100 µL of nuclease free IDTE buffer (1X TE buffer, pH 7.5), respectively, to obtain 50 µM solutions of each. To form active gRNA complexes, an equimolar concentration of crRNA1 and tracrRNA (i.e. 2 µL each at a 50 µM stock concentration) were hybridized by heating up to 95 °C in a heat block followed by cooling down to room temperature (RT) for 10 min on bench top. To this 4 µL gRNA, 1 µL of 61 µM (10 µg) of Alt-R S.p. Cas9 Nuclease V3 or Alt-R HiFi S.p. Cas9 Nuclease V3 was added along with 2 µL of 1X PBS buffer (pH 7.4). The solution was mixed by pipetting followed by short centrifugation and incubated at RT for 10 min to form an RNP complex. Similarly, for Alt-R S.p. Cas9 D10A Nickase V3, 1 µl each of 50 µM crRNA1 and crRNA2 were combined with tracrRNA as described above to form gRNA and RNP complex. In the case of Cas12a enzymes, the custom synthesized crRNA3 for Alt-R A.s. Cas12a V3 and LbCas12a V3 were dissolved with 40 µL of nuclease free IDTE buffer (1X TE buffer, pH 7.5). To form RNP complexes, 2 µL of 50 µM AsCas12a or LbCas12a crRNA3 were combined with 1 µL from the 10 µg/µL (63 µM) Alt-R A.s. Cas12a V3 or LbCas12a, respectively, to form RNP complexes as previously described.

Gold coating with RNP complexes

To coat the RNP complexes onto 0.6 µM gold particles (Bio-Rad cat #1652262), gold particle solution was prepared as previously described by Banakar and Wang (2019). To a tube of sonicated 1X-gold solution (0.75 mg of gold in 25 µL H_2O-prewarmed to RT), 7 µL or 5 µL of RNP complexes of Cas9 and Cas12a, respectively, were transferred. To impart hygromycin resistance and enrich for transformed cells, 2 µL of pCAMBIA1301 (500 ng) plasmid was added and mixed well by pipetting up and down. To this mixture, 2 µL of water-soluble cationic lipid TransIT-2020 (Mirus Bio LLC, Madison, WI) was added and mixed, and then the solution was solution was incubated on ice for 10 min. Tubes were centrifuged at 8000 rpm for 30s, the supernatant was discarded, and the pellet was dissolved in 20 µL of sterile nuclease free double distilled water (sdH_2O) and sonicated 10 s to homogenize the solution. Two macrocarrier discs were prepared by pipetting 10 µL of the solution onto the pre-sterilized macrocarriers in the laminar flow hood. Macrocarriers were then air dried in the hood for about 20 to 30 min until a dry yellow powder was visible.

Biolistic co-delivery of RNP and pCAMBIA1301 in rice

Rice (*Oryza sativa* L. cv Nipponbare) was grown in a growth chamber (12:12 light:dark) at Iowa State University, Ames, IA, as previously described by Banakar and Wang, 2019. Seeds were harvested and placed in an envelope and air dried at 37 °C for 3 days immediately before use for transformations. Seed culture to obtain scutellar derived embryos, osmotic treatment, post osmotic recovery, selection, regeneration and rooting was performed as previously described (Banakar and Wang, 2019; Banakar, 2016; Banakar et al, 2017; Sudhakar et al., 1998). Briefly, seeds were germinated on Murashige and Skoog media with 2 mg/L, 2,4-D for 6 days (27 °C, dark). On the 6th day scutellum-derived embryos were extracted and placed on MS media with 2 mg/L 2,4-D. On the seventh day scutellar rice embryos were plated on osmoticum medium with the scutellum facing up for 4 hours before bombardment. The BioRad Biolistic® PDS-1000/He particle delivery system was used to co-deliver RNPs and pCAMBIA1301 using 900 PSI rupture discs and a 6 cm target distance. For each enzyme two technical replicates of 30 embryos each was bombarded. Post biolistic delivery embryos were kept on the same osmoticum media for 16 hours (27 °C, dark) before transfer to resting media (MS media with 2 mg/L 2,4-D) for 24 hours (27 °C, dark). This was followed by two rounds of selection (15 days each) on MS selection media (2 mg/L 2,4-D) containing 50 mg/L hygromycin. Surviving calli were transferred to MS regeneration media with BAP (2.5 mg/L), and NAA (0.25 mg/L) and hygromycin (50 mg/L). Surviving tissues from regeneration media were transferred to ½ strength MS media supplemented with the full strength of B5 vitamins. Through the tissue culture process tissue derived from a single embryo was considered to be clonal for transgenes. Hence, all the tissue derived from a single embryo in the rooting media plates was collected and pooled as single clone, and stored in -80 °C until further use. When clones were rooted green plantlets, seedlings were transferred to soil.

DNA extraction

Genomic DNA was isolated from transgenic calli as previously described by Edwards et al, 1991 with slight modification. Tissues were ground to a fine powder in liquid nitrogen using a pestel and mortar. For each sample 50 mg of powdered tissue was used for DNA extraction. Five hundred µl of extraction buffer (200 mM Tris buffer, pH 7.5, 250 mM NaCl, 25 mM Na_2•EDTA, and 0.5% sodium dodecyl sulfate [SDS]) was added, and the material was vortexed. Following a phenol/chloroform extraction, a chloroform extraction, and precipitation with 0.7 volumes of isopropanol, DNA pellets were resuspended in 50 µl Milli-Q H_2O and quantified using a NanoDrop spectrophotometer.

Next Generation Sequencing analysis

The genomic region surrounding the target sites was amplified by using 50 ng of template DNAs and forward primer (TTGCAGACGCTCTTGCG) and reverse primer (CAAAGCTCACATGCTGACTACT). The primers contained 'tails' to add sample-unique P5 and P7 indexes for Illumina sequencing in two rounds of PCR. PCR amplicons were sequenced on an Illumina MiSeq instrument (v2 chemistry, 150 bp paired end reads) (Illumina, San Diego, CA, USA). Data were analyzed using a custom-built pipeline. Data were demultiplexed (Picard tools v2.9; https://github.com/broadinstitute/picard) and forward and reverse reads were merged into extended amplicons (flash v1.2.11) (Magoc and Salzberg, 2011). Reads with any base quality score <10 were filtered out. At each target, editing was calculated as the percentage of total reads containing an INDEL within a 10 bp window of the cut site. For each transgenic clone reads with >5% editing was considered as edited.

References

Banakar R (2016) Mechanisms controlling selective iron and zinc biofortification of rice. https://repositori.udl.cat/handle/10459.1/64285

Banakar R, Alvarez Fernández Á, Abadía J, Capell T, Christou P (2017) The expression of heterologous Fe (III) phytosiderophorer transporter HvYS1 in rice increases Fe uptake, translocation and seed loading and excludes heavy metals by selective Fe transport. Plant Biotechnol J 15:423-432

Banakar R, Wang K (2019) Biolistic transformation of Japonica rice varieties. In: Biolistic DNA Delivery in Plants. S. Rustgi, H. Luo (eds), Springer, New York, NY. In press.

Edwards K, Johnstone C, Thompson C (1991) A simple and rapid method for the preparation of plant genomic DNA for PCR analysis. Nucleic Acids Res 19: 1349

Magoc T, Salzberg SL (2011) FLASH: fast length adjustment of short reads to improve genome assemblies. Bioinformatics 27:2957-2963

Sudhakar D, Duc LT, Bong BB, Tinjuangjun P, Maqbool SB, Valdez M, Jefferson R, Christou P (1998). An efficient rice transformation system utilizing mature seed-derived explants and a portable, inexpensive particle bombardment device. Transgenic Res 7:289– 294
